# Mobile health in the management of type 1 diabetes: a systematic review and meta-analysis

**DOI:** 10.1186/s12902-019-0347-6

**Published:** 2019-02-13

**Authors:** Xuemei Wang, Wei Shu, Jian Du, Maolin Du, Peiyu Wang, Mingming Xue, Huiqiu Zheng, Yufeng Jiang, Shaohua Yin, Danyan Liang, Ruiqi Wang, Lina Hou

**Affiliations:** 1 Department of Nutrition and Food Hygiene, School of Public Health, Peking University Health Science Center, Beijing, 100191 China; 2 Department of Health Statistics, School of Public Health, Inner Mongolia Medical University, Hohhot, 010110 China; 3 Clinical Center on TB Control, Beijing Tuberculosis and Thoracic Tumor Research Institute/Beijing Chest Hospital, Capital Medical University, Beijing, 101149 China; 4 Department of Social Medicine and Health Education, School of Public Health, Peking University Health Science Center, Beijing, 100191 China; 5 School of Basic Medicine, Inner Mongolia Medical University, Hohhot, 010110 China; 6 Diabetes Centre, The 306th Hospital of PLA, Beijing, 100101 China

**Keywords:** Effectiveness of mobile health, HbA1c level, Mobile health intervention, Systematic review, Type 1 diabetes

## Abstract

**Background:**

As an insulin-dependent disease, type 1 diabetes requires paying close attention to the glycemic control. Studies have shown that mobile health (mHealth) can improve the management of chronic diseases. However, the effectiveness of mHealth in controlling the glycemic control remains uncertain. The objective of this study was to carry out a systematic review and meta-analysis using the available literature reporting findings on mHealth interventions, which may improve the management of type 1 diabetes.

**Methods:**

We performed a systematic literature review of all studies in the PubMed, Web of Science, and EMbase databases that used mHealth (including mobile phones) in diabetes care and reported glycated hemoglobin (HbA1c) values as a measure of glycemic control. The fixed effects model was used for this meta-analysis.

**Results:**

This study analyzed eight studies, which involved a total of 602 participants. In the meta-analysis, the fixed effects model showed a statistically significant decrease in the mean of HbA1c in the intervention group: − 0.25 (95% confidence interval: − 0.41, − 0.09; *P* = 0.003, *I*^2^ = 12%). Subgroup analyses indicated that the patient’s age, the type of intervention, and the duration of the intervention influenced blood glucose control. Funnel plots showed no publication bias.

**Conclusions:**

Mobile health interventions may be effective among patients with type 1 diabetes. A significant reduction in HbA1c levels was associated with adult age, the use of a mobile application, and the long-term duration of the intervention.

## Background

Type 1 diabetes mellitus (T1DM) is a metabolic disease characterized by hyperglycemia [1]. This disease, also referred to as insulin-dependent diabetes, occurs in all age groups, but especially in children and adolescents [2]. With an increasing worldwide incidence of approximately 3% to 4% a year [3]. In the United States, the number of young patients with T1DM has been predicted to increase by 23% over the next 40 years [4]. Although T1DM accounts for only 5–10% of all cases of diabetes, the prevalence of T1DM is increasing in most countries around the world [5]. The significant effect that poor glycemic control can have on the health of patients with T1DM [6], includes failure to achieve adequate levels of glycemic control ultimately results in damage to a wide range of organs, most notably the eyes, kidneys, heart, blood vessels, and nerves [7]. With the growing number of individuals with T1DM, improving diabetes care to decrease the health and economic burden caused by the disease is viewed globally as an important goal [1, 8].

Studies have shown that continuous self-health management strategies positively impact many aspects of T1DM, including preventing complications and improving both metabolic control and quality of life [9–11]. However, the diabetes education provided in clinics and hospitals is limited, and the availability of this education is severely restricted. The market penetration of mobile phones is extensive, and they currently meet a variety of user needs [11–13]. In 2015, it was reported that 88% of American teens either owned or had access to a mobile phone, compared with 45% in 2004 [14]. At present, mobile phones, apart from their recreational function, are becoming instruments of patient education and support and are also helpful for health care professionals [15, 16]. A number of studies have shown that mobile phone, telehealth and similar movements with increasing popularity as the tools to aid persons with diabetes in the managing of their condition while lowering costs [17, 18]. New mHealth technology can also improve the quality of life of diabetic patients [19, 20]. The impact of mHealth interventions on the control of HbA1c levels in type 2 diabetes is clinically significant and has been well documented [21, 22]. Toma et al. [23] investigated the effectiveness of online social networking services or mobile phone use as a management intervention for patients with diabetes, finding that social networking services interventions beneficially reduced Hemoglobin A1c (HbA1c) when compared with the non user. This finding was confirmed by sensitivity analysis; there was a significant reduction in HbA1c in the intervention group (weighted mean difference [WMD] = 0.46%; 95% confidence interval [CI]: 0.58–0.34). A number of studies have evaluated the use of mHealth in the management of patients with T1DM; however, these studies’ results have varied [24–26], and the effectiveness of mHealth on glycemic control remains uncertain.

Therefore, the objective of the present study was to conduct a systematic review and meta-analysis of published studies to evaluate the efficacy of interventions including mHealth compared with other interventions to control HbA1c levels in populations of children and adults.

## Methods

### Search strategy

Studies published in English were identified by searching PubMed, Web of Science, and EMbase. In addition, we searched within the reference lists of identified papers. We searched for studies published through June 2016 using combinations of the following search terms: “mHealth,” “mobile health,” “text-messaging,” “mobile application,” “type 1 diabetes,” “diabetes mellitus,” and “randomized controlled trial.” These search terms were connected or used alone using “and” or “or,” and the search strings were developed according to the characteristics and requirements of each database and the particular search engines employed.

### Inclusion and exclusion criteria

The authors made a selection from the identified articles for this review. We defined patients with T1DM as those who had been diagnosed by a physician, and we selected interventions that lasted more than three months (the time the experimental group used the mHealth programme) [27]. Participants with T1DM were included regardless of gender, age, race, or nationality. To be included in this study, the patients had to have the reading and writing skills necessary to complete their medical histories and the questionnaires independently. Additional inclusion criteria were as follows: (1) randomized controlled trials with mHealth as an intervention (i.e., the interventions included mobile applications or text messages); (2) the inclusion of a comparison of standard therapies (i.e., receiving the standard educational approach, without mobile applications or text messages); (3) reporting HbA1c as an outcome, with values measured at both baseline and at the end of the study for each group; and (4) written in English. Studies were excluded if they (1) included patients with Severe diabetic complications(Diabetic foot, Diabetic heart disease.etc); (2) had mixed patient populations (type 1 and type 2 diabetics); (3) conducted interventions by voice via telephone; (4) covered only the use of insulin pumps, artificial pancreas, or continuous glucose monitoring equipment; (5) duplicate publications of the same data set; (6) drew upon original data that was not available; (7) were limited to pregnant women or other special populations with T1DM. All analyses for the present study were based on previous published research; thus, no ethical approval or patient consent were required.

### Data extraction and quality assessment

Two reviewers independently scanned the electronic records to identify potentially eligible trials. A standard data extraction sheet was used to extract and sort these trials independently. All discrepancies were resolved in discussion with a third reviewer. We included studies that met our inclusion criteria. The variables extracted from the studies included country of origin; year of publication; number of participants; participants’ age, sex, diabetes duration, and HbA1c at baseline and at follow-up; the intervention; and the follow-up time. We reported duplicate publications of the same data set only once. For studies with more than one intervention group, we considered the most intensive intervention to be the experimental one. Following the search strategy described above, no studies were included for which the necessary data from the original study were not reported.

The risk of bias was assessed using the Cochrane Collaboration tool [28]. The studies were rated according to five predefined categories: (1) random sequence generation; (2) quality of allocation concealment; (3) quality of blinding; (4) freedom from incomplete data; and (5) freedom from selective reporting. The risk of bias in each area was scored as high, low, or unclear.

### Data synthesis and analysis

We determined that the primary outcomes used the fixed effects model and weighted mean difference (WMD). Analysis was conducted using Review Manager Version 5.3 for Windows (The Cochrane Collaboration, Software Update, Oxford, UK). Heterogeneity was measured using Cochrane’s Q and the *I*^*2*^ index to test whether the studies were homogeneous, the *p* value of the Q-test and the *I*^*2*^ index were set at 0.10 and 50%, respectively. If *P* > 0.10 and *I*^*2*^ < 50%, the results of homogeneity were considered good, and the fixed effects model was used for analysis. Otherwise, the random effects model was used. The meta-analysis test level was set at *α* = 0.05. Subgroup analyses further explored the effects of different ages, types of interventions, and intervention durations on the study results. A funnel plot was used to detect publication bias.

## Results

### Literature screen

**Fig. 1:**
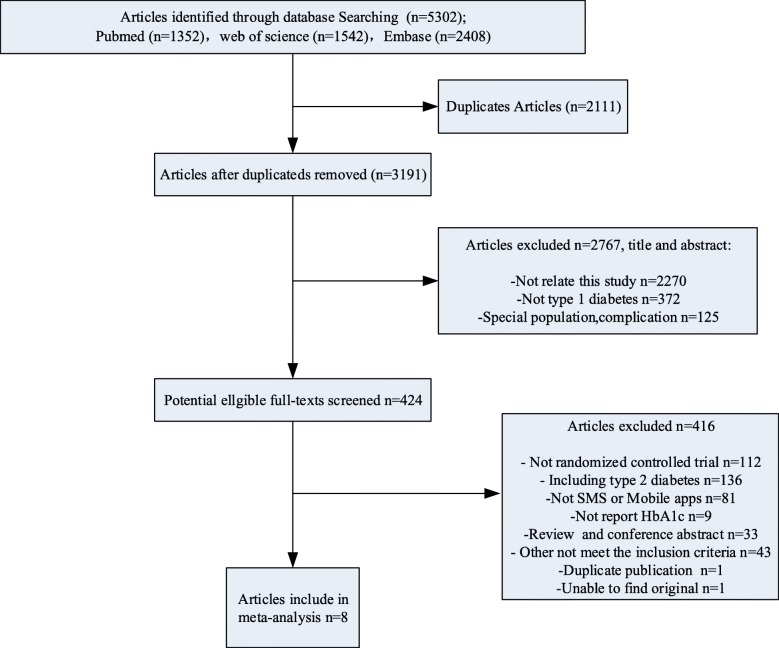
Flow diagram for study selection according to the Preferred Reporting Items for Systematic Reviews and Meta-analyses (PRISMA) guidelines

This systematic review was structured according to the Preferred Reporting Items for Systematic Review and Meta-Analyses guidelines [29]. From 5302 citations, 424 publications were identified as potentially eligible studies, and the full text of these publications was retrieved and assessed. Eight studies met the inclusion criteria [24–26, 30–34]. Details of the screening process and results are presented in Fig. 1.

### Study characteristics

The studies that were included in the systematic review are listed in Table 1. All of the eight eligible studies were published from 2005 to 2016. They included a total of 638 patients, 325 of whom were randomly assigned to intervention groups; 313 were assigned to control groups. A total of 578 patients (90.5%) completed the respective intervention studies, there were 293 people in the intervention group and 285 in the control group. A total of 602 people were analyzed. And, for the six references that calculated the sample size, the power was greater than 80%. There was no statistically significant difference in the rate of loss to follow-up between the intervention group and the control group. Two studies were conducted in the United States, five in Europe, and one in Australia. The shortest study was 3 months in duration, and the longest was 12 months. Three studies had durations of 3–5 months, and five studies had durations longer than 6 months. In three studies, the intervention was only use mobile phone text messages, while the remainder used mobile phone applications.

**Table 1:** Characteristics of the studies included in the meta-analysis

Author Year, Country	Study types	Number of participants (I/C)	Age years (I/C)	Sex(M/F)		Diabetes duration (I/C)	Intervention type	HbA1c at baseline (I/C)	HbA1c at follow-up (I/C)	Follow-up (months)
				I	C					
Han, Y 2015, USA	RCT	20 (10/10)	13.8±1.874/12.9±2.183	4/6	5/5	6±4.662/7.3±3.164	mobile text message	8.60±0.867/8.78±0.728	8.23±1.370/8.57±0.786	3-4
Berndt 2014, Germany	RCT	68 (34/34)	12.9±2.0/13.2±2.9	21/13	20/14	5.0±3.7/5.3±4.0	mobile applications	8.84±1.71/8.96±2.23	8.12±1.10/7.99±1.26	3
Charpentier 2012, France	RCT	121 (60/61)	32.9±11.7/36.8±14.1	23/37	21/40	17.6±8.9/16.9±10.5	mobile applications	9.19±1.14/8.91±0.90	8.63±1.07/9.10±1.16	6
David 2009, USA	RCT	40 (22/18)	17.7±3.0/18.2±2.3	9/13	9/9	9.0±4.2/9.5±5.6	mobile text message	8.9±1.7/8.6±0.9	8.7±1.5/8.8±0.9	3
Franklin 2008, Scotland	RCT	60 (33/27)	14.1 (11.7-15.6)/12.7 (10.5-14.8)	15/18	17/10	4.8 (2.6-8.6)/3.2 (1.7-6.7)	mobile text message	9.8±1.8/10.2±1.6	10.1±1.7/10.3±1.7	12
Rossi 2013, Italy	RCT	127 (63/64)	38.4±10.3/34.3±10.0	29/34	31/33	16.2±10.0/15.0±8.4	mobile applications	8.4±0.79/8.5±0.8	7.9±0.79/8.1±0.8	6
Kirwan 2013, Australia	RCT	72 (36/36)	35.97±10.67/34.42±/10.26	19/17	9/27	19.69±9.64/18.19±9.77	mobile applications	9.08±1.18/8.47±0.86	7.80±0.75/8.58±1.16	9
Rossi 2010, Italy	RCT	130 (67/63)	35.4±9.5/36.1±9.4	30/37	26/37	17.1±10.3/15.8±10.7	mobile applications	8.2±0.8/8.4±0.7	7.8±0.8/7.9±1.1	6

*Note. I/C* intervention group/Control group, *RCT* randomized controlled trial, *IQR* interquartile range, *M/F* male/female

Values are Mean ± SD for continuous normal distribution, Median (IQR) and Median (range) for continuous skewed distribution

Mobile text message: Using short message to intervene patients; Mobile applications: Use mobile phone applications to interact with patients and educate patients

Table 1 describes the basic features of the literature

### Outcomes

#### Comparison of HbA1c levels at baseline and follow-up

**Fig. 2:**
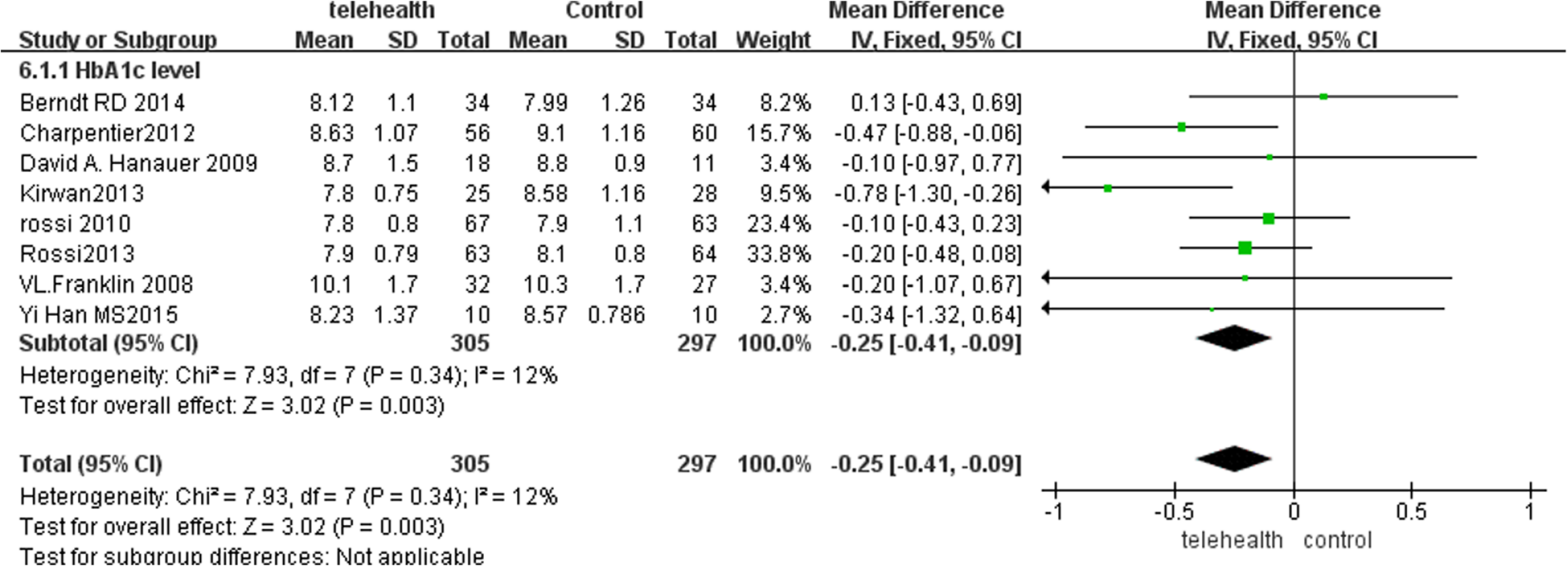
Forrest plots demonstrating the effect of mobile health on HbA1c

There was no difference in HbA1c between the intervention and control groups at baseline, the mean of HbA1c (95% confidence interval) is (0.00 [− 0.15, 0.14]; *P =* 0.97). There was a significant reduction in the follow-up HbA1c in the intervention group, compared with their baseline HbA1c (− 0.54 [− 0.80, − 0.28]; *P =* 0.0001). In contrast, the standard-care group showed no significant difference between baseline and follow-up HbA1c (− 0.18 [− 0.45, 0.08]; *P =* 0.17), obtained before and after the intervention. There was a significant difference in the pooled HbA1c change from baseline between the mHealth and standard-care groups (− 0.25 [− 0.41, − 0.09]; *P = 0.003*) (Fig. 2). Regarding statistical heterogeneity, the variability in effect estimates (I^2^ = 12%, *P* = 0.34) showed the homogeneity of the intervention effects across the primary studies was good. Therefore, the fixed effects model was used for the primary data analysis.

### Subgroup analyses of different ages, types of intervention, and intervention durations

To assess the effects of different characteristics on the intervention, we performed subgroup analyses grouped by age, type of intervention, and intervention time. The results showed that adult patients, using mobile applications for patient management, the implementation of a longer period of intervention on patients with HbA1c control is effective (Table 2).

**Table 2:** Subgroup analysis of reduction in HbA1c

Subgroups	Studies (n)	Effect size (%)	I^2^ (%)	P value for heterogeneity in subgroups
Age				
Teenagers	4	-0.05[-0.43,0.33]	0	0.84
Adult	4	-0.29[-0.47,-0.11]	48	0.12
Intervention ways				
Text-message	3	-0.20[-0.73,0.32]	0	0.94
Mobile application	5	-0.25[-0.42,-0.08]	49	0.10
Duration of intervention				
<6 months	3	-0.01[-0.44,0.41]	0	0.70
>=6 month	5	-0.29[-0.46,-0.11]	32	0.21

Table 2 describes the results of a combination of subgroup analyses

1. Age

Because T1DM is managed differently in different age groups [35], we analyzed a youth group and an adult group. We found that the change in HbA1c in the youth group was not significant (− 0.05 [− 0.43, 0.33]; *P = 0.80*). In the adult group, however, there was a significant change in HbA1c (− 0.29 [− 0.47, − 0.11]; *P =* 0.001).

2) Types of interventions

For the subgroup analyses of different types of interventions, intervention methods were classified into two categories: text messages (including automated Internet text messages and short message service [SMS]) or mobile applications about diabetes self-management. The use of SMS as an intervention was not associated with a significant decrease in HbA1c (− 0.20 [− 0.73, 0.32]; *P* = 0.44), whereas HbA1c levels were better controlled with the use of mobile phone applications (− 0.25 [− 0.42, − 0.08]; *P* = 0.003).

3) The duration of the interventions

We created two subgroups: interventions of less than 6 months and interventions of more 6 months. The results showed significantly decreased HbA1c levels in the group with interventions of more than 6 months (0.29 [− 0.46, − 0.11]; *P* = 0.001). In the group with interventions of less than 6 months HbA1c did not significantly change (− 0.01[− 0.44, 0.41]; *P =* 0.95).

### Risk of bias

**Fig. 3:**
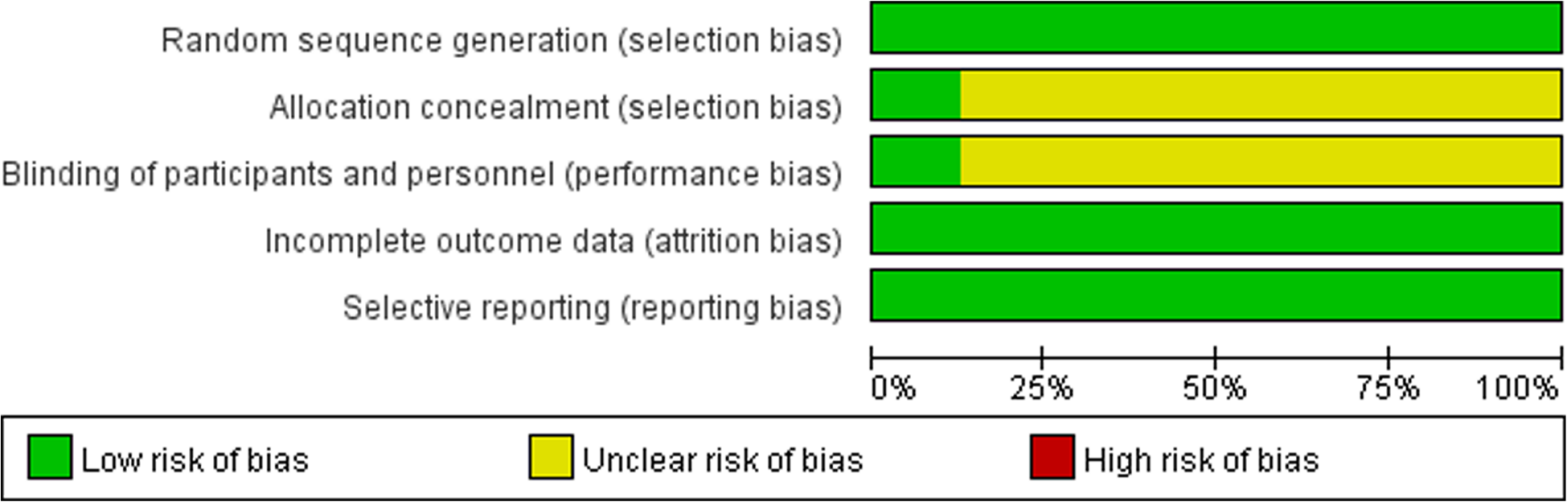
Cochrane risk of bias graph

**Fig. 4:**
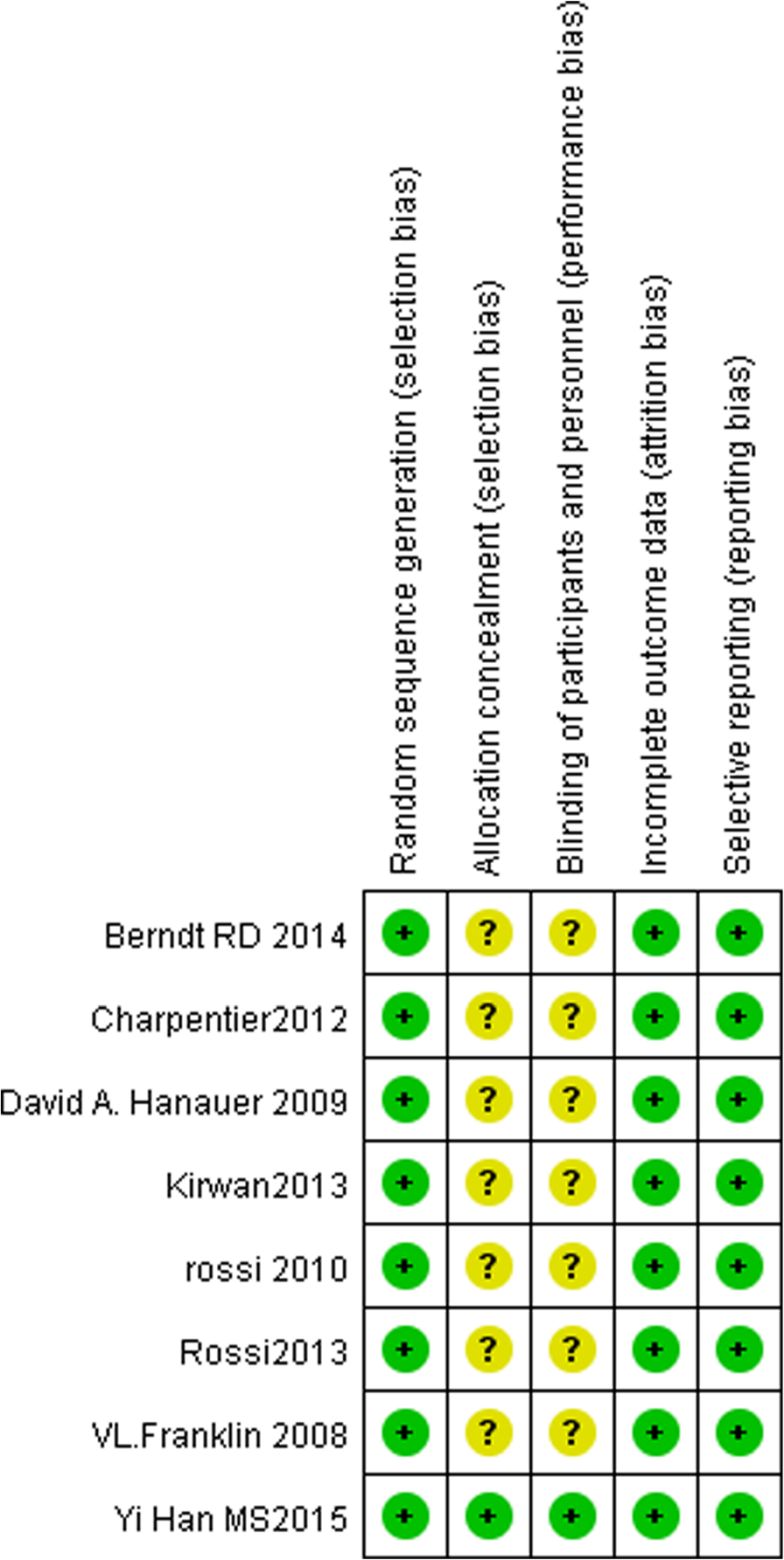
Risk of bias summary

The risk of bias of the included studies is shown in Figs. 3 and 4. All of the studies were randomized and therefore at a lower risk of bias. This was because of the nature of the intervention; some of the studies could not use blinding, which influenced the confidence of the synthetic results. All of the studies were free of incomplete outcome data, and all of the trials were free of selective outcome reporting.

### Publication bias

**Fig. 5:**
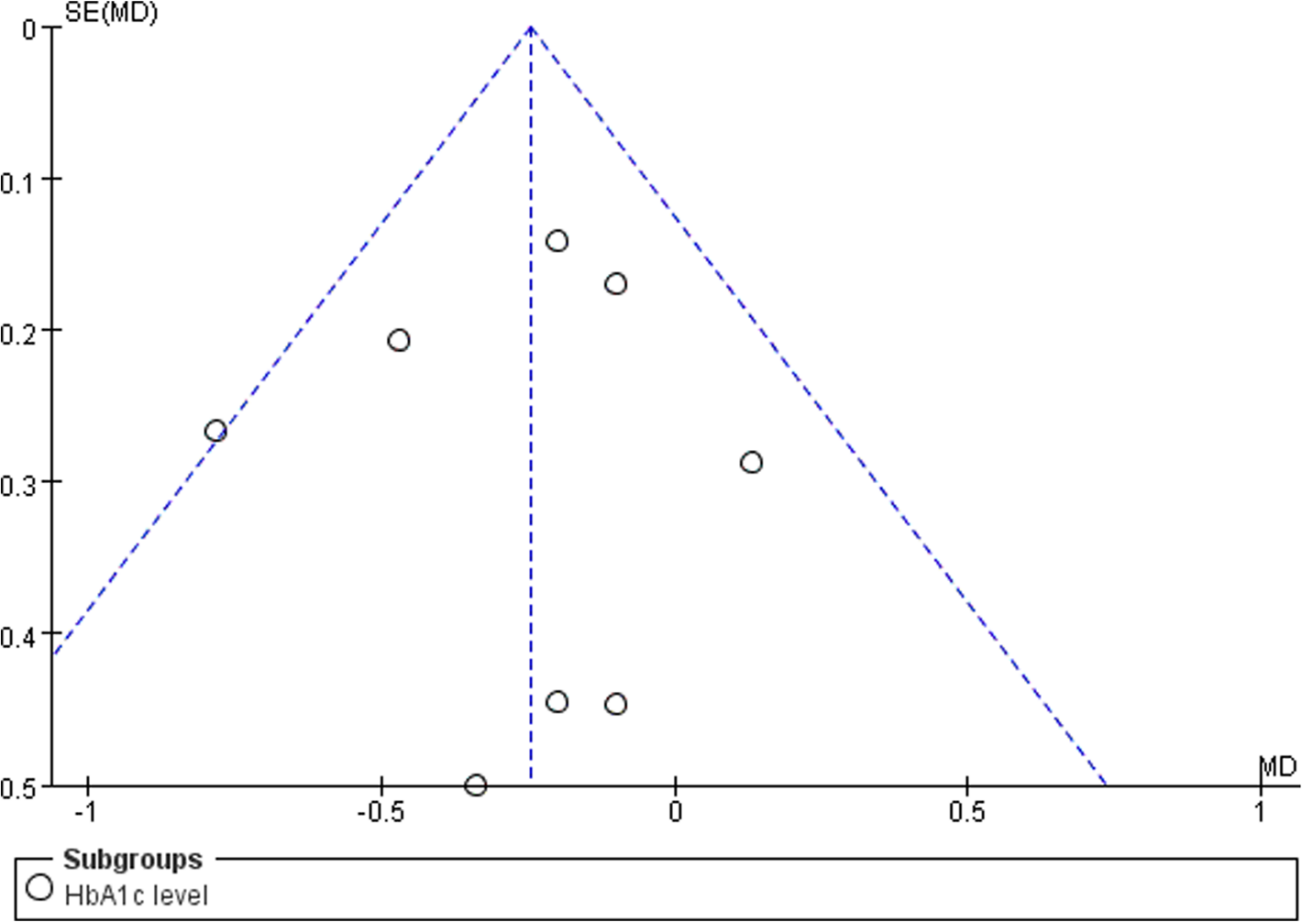
Funnel plot of comparison

Publication bias was assessed by a funnel plot (Fig. 5). Each study was symmetrically distributed on both sides. No significant publication bias was observed.

## Discussion

Maintaining a healthy lifestyle in patients with T1DM is fundamental to their health status and welfare. The main objective of the present study was to provide the most recent information on mHealth, and the findings are based on studies conducted in different countries. Among the reviewed studies, all applied randomized controlled designs, which enhanced the comparability of the outcomes. In the fixed effects model used in the meta-analysis, heterogeneity less than 50%, indicating that the results are relatively reliable. The results of this meta-analysis showed that using mHealth interventions reduced HbA1c relative to no mHelath control groups. Our results are consistent with research on the management of chronic diseases [36, 37], which has demonstrated improved medication adherence with the use of text messages or mobile applications [38]. However, it should be noted that some studies have found no significant difference in HbA1c between intervention and control groups. This may be due to the greater emphasis in developed countries on primary health care for diabetics, where a higher health consciousness might have been a confounding factor [39]. Therefore, mHealth can still improve a patient’s understanding of a disease process and self-management strategies [25]. Skrovseth [40] has demonstrated that the effective implementation of mHealth has a significant positive impact on blood glucose control. In addition, in some studies, glycosylated hemoglobin also decreased significantly in patients who did not receive the intervention (control group) [25, 32, 33]. This suggests that although routine intervention can also help patients control their HbA1C, it is less effective than the mhealth group.

Subgroup analyses found that the impact of mHealth differed for teenage and adult patients with T1DM. mhealth interventions had a significant positive impact among adults, whereas the effects of these interventions were subtler for younger people. This is consistent with the results of several previous systematic reviews [41]. The management of children with T1DM is complicated by multiple factors that must be taken into consideration, such as growth, activity, diet, insulin enhancement, and psychological factors [42]. Additionally, as children mature into adolescents and then adults, changes in growth hormone secretion result in insulin resistance [43, 44]. Furthermore, although the patients who were enrolled in our study were required to be able to read and write, compared with adults, adolescents’ self-management ability is poor, and their understanding of health education is not clear enough [39]. Poor compliance may affect glycemic control [45]. Although HbA1c were not significantly different following the intervention in the adolescent group, both quality of life and treatment compliance were greatly improved [25, 26]. Others have found that teenagers are prone to accept this form of education [46]. This also suggests that mhealth treatment has potential but needs to be refined by teenagers and can be cost-effective as a means of intervention. In-depth study is required to determine an effective self-management model for minors.

Our subgroup analyses found that using mobile applications was more effective than using text messages. Text message interventions were associated with lower costs and increased ease of operation, providing a wide range of intervention opportunities [47]. However, in contrast to the mobile application, text messaging lacks two-way communication. Text messaging also has a simpler intervention content and a lower rate of patient feedback. Studies have shown that the use of mobile applications by diabetic patients is increasing, indicating that patients with diabetes are interested in using these methods to improve their self-management [48]. Compared with other methods, mobile applications can provide better education and more timely feedback [49]. Some mobile applications also support the real-time monitoring of blood glucose [48]. The future direction of mhealth should strive to facilitate the expansion of effective health management practices among the general population. Therefore, studies should further explore the use of mhealth to improve the level of patient self-management the most effective method.

The duration of the intervention may be an important factor in determining whether mHealth is effective. Our subgroup analysis showed that the decrease in HbA1c was significant in groups exposed to longer continuous intervention time. This indicates that longer intervention time periods lead to better glycemic control. Comparing groups exposed to interventions of different durations, Kirwan et al. [24] found that blood glucose control in patients with 9 months of intervention was better than in those with 3 months. This is likely because diabetes is a chronic disease with a slow process of control. HbA1c reacts to blood glucose levels for nearly 2 months. Therefore, short-term interventions may not result in significant changes in HbA1c levels. In summary, we need to pay attention to the duration of the intervention.

### Limitations

Despite the positive findings of this study, a number of limitations should be considered when interpreting the results: First, although the findings from the reviewed studies showed the use of mobile phones to be promising for improving T1DM management, some of these studies had small sample sizes. Especially in the subgroup analyses, small numbers may be lead to false positive results. Therefore, future studies with large sample sizes are needed to determine whether the increased patient–provider communication with mHealth has a significant impact on clinical outcomes and public health. Second, it is possible that our search for relevant literature for inclusion in the current review paper may have overlooked some publications. If so, this could cause selection bias. Further studies should be conducted to confirm the present findings. Third, the studies included in this review had different characteristics; these factors can lead to heterogeneity and influence the reliability of the results. Fourth, Glycosylated hemoglobin (HbA1c) is a gold standard to measure blood glucose control, which can effectively reflect the past blood glucose control in patients with diabetes mellitus. Part of the literature included in this study had a relatively short intervention time, which may have an impact on the results.

## Conclusions

In summary, this study found that the use of mobile health for patients with T1DM positively impacted disease management and improved HbA1c levels in certain subgroups. However, because of the limitations inherent in both the quantity and quality of the included studies, high quality, multicenter, randomized controlled trials with large sample sizes are needed to substantiate these results.

## Abbreviations

CI: Confidence interval
HbA1c: Hemoglobin A1c
mHealth: Mobile health
SMS: Short message service
T1DM: Type 1 diabetes mellitus
WMD: Weighted mean difference
